# Apolipoprotein B correlates with intra-plaque necrotic core volume in stable coronary artery disease

**DOI:** 10.1371/journal.pone.0212539

**Published:** 2019-02-19

**Authors:** Takayuki Ohwada, Takayuki Sakamoto, Yuki Kanno, Sayoko Yokokawa, Kazuaki Amami, Kazuhiko Nakazato, Yasuchika Takeishi, Kenichi Watanabe

**Affiliations:** 1 Fukushima Red Cross Hospital, Department of Cardiology, Fukushima City, Japan; 2 Fukushima Medical University, Department of Cardiology, Fukushima City, Japan; Nagoya University, JAPAN

## Abstract

**Objective:**

To determine the relationship between plaque composition and low-density lipoprotein cholesterol (LDL-C), high-density lipoprotein cholesterol (HDL-C), apolipoprotein B (Apo-B), and Apo-A1 using virtual-histology intravascular ultrasound (VH-IVUS).

**Methods:**

We assessed plaque composition in patients with stable coronary artery disease (SCD) admitted to our hospital for percutaneous coronary intervention (PCI) between November 1, 2012, and March 10, 2015. Before PCI, fibrous (FI), fibrofatty (FF), necrotic core (NC), and dense calcium (DC) regions were evaluated using VH-IVUS, and the contributions of each to the culprit lesion volume were recorded. Plasma LDL-C, HDL-C, Apo-B, and Apo-A1 levels were assessed before PCI. The relationship between the regions on VH-IVUS and plasma lipid levels was assessed. Patients were categorized into low Apo-B (LAB) and high Apo-B (HAB) groups, based on the overall cohort median Apo-B level.

**Results:**

We enrolled 115 patients (median Apo-B, 91 mg/dL, male n = 88) with 57 and 58 patients in the LAB (Apo-B ≤ 90 mg/dL) and HAB (Apo-B ≥ 91 mg/dL) groups, respectively. Vessel, plaque, and %NC volumes were significantly greater in the HAB group than in the LAB group. The %FI, %FF, and %DC volumes were similar in both groups. In all 115 patients, the %NC volume correlated with LDL-C (r = 0.2353, P = 0.0114) and Apo-B (r = 0.2487, P = 0.0074) but not with HDL-C and Apo A-1. The high-sensitivity C-reactive protein level tended to be higher in the HAB group than in the LAB group. Multiple regression analysis showed that being male, Apo-A1, and Apo-B were significant predictors of %NC volume extent.

**Conclusions:**

Elevated Apo-B level was related to the %NC in target coronary artery lesions in SCD patients, suggesting a role of Apo-B as a biomarker of unstable plaque in this population.

## Introduction

Low-density lipoprotein cholesterol (LDL-C), total cholesterol (TC), and high-density lipoprotein cholesterol (HDL-C) are standard biomarkers used to predict adverse cardiovascular events in patients with ischemic heart disease. [1, 2] However, recent reports indicate that apolipoprotein B (Apo-B) has a greater predictive value than LDL-C. [1–4]

Using virtual-histology intravascular ultrasound (VH-IVUS), the PROSPECT study demonstrated that the necrotic cores (NCs) of thin-cap fibroatheromas are sites at which processes associated with adverse cardiovascular events occur. [5] Intensive lipid-lowering by statins can lead to atheromatous plaque stabilization or regression through various mechanisms, such as the reduction of inflammation and lipid accumulation in the core. Furthermore, statin therapy alters the plaque composition in coronary arteries, although the mechanism for this change is controversial. [6–8]To date, no study has used IVUS to evaluate the associations of LDL-C and Apo-B with atheroma composition. Further, the effects of lipoproteins and apolipoproteins on plaque composition in patients with stable coronary artery disease (SCD) are unclear and whether these proteins affect plaque vulnerability to rupture is not known. Conventional gray-scale IVUS images are generated using the amplitude of the radiofrequency (RF) signal. However, the frequency and power of the signal differ between tissues, regardless of similarities in the amplitude. Therefore, gray-scale IVUS has limited value in the accurate identification of specific plaque components. Analysis of IVUS radiofrequency backscatter enables a more detailed characterization of plaque morphology and tissues, and provides insight into the features of vulnerable plaque. [9]

Therefore, we used VH-IVUS to investigate the relationship between Apo-B and atheroma composition in patients with SCD. Our hypothesis was that Apo-B could be a potential biomarker for acute coronary syndrome.

## Materials and methods

The Fukushima Red Cross Hospital Ethics Committee approved this study, and all study participants provided written informed consent for participation before enrollment.

### Study population

We prospectively evaluated 334 consecutive patients with SCD that visited our hospital for percutaneous coronary intervention (PCI) between November 1, 2012 and March 10, 2015, for study eligibility. Forty patients selected medical therapy. A total of 294 patients who underwent diagnostic coronary angiography (CAG) and had coronary artery stenosis greater than 75% were eligible for the study. PCI indications were evaluated according to the Japanese Society of Cardiology guidelines for elective PCI in patients with SCD. [10] We excluded patients who did not consent to participate (63 patients) and those who had no significant stenosis (50 patients), chronic total occlusion (18 patients), and restenosis of a prior stent (20 patients). After obtaining consent, we enrolled 143 patients and performed PCI; IVUS was performed in all enrolled patients. We excluded patients when IVUS could not be completed without balloon angioplasty because of a severely stenosed, tortuous, or heavily calcified culprit lesion (16 patients). Patients with VH-IVUS images of inadequate quality for analysis were also excluded (12 patients). The remaining 115 patients underwent plasma Apo-B measurements and were categorized into the low Apo-B (LAB) or high Apo-B (HAB) groups on the basis of their overall cohort median Apo-B level.

### IVUS image acquisition

IVUS examinations of the culprit lesions were performed before PCI. A phased-array, 20-MHz, 3.2-F IVUS catheter (Eagle Eye, Volcano Corporation, Rancho Cordova, CA, US) was placed in the distal coronary artery and pulled back to the aorto-ostial junction using a motorized catheter pull-back system set at 0.5 mm/s (Eagle Eye, Volcano Corporation). The gray-scale IVUS and captured radiofrequency data were written onto a DVD-R.

### Gray-scale and VH-IVUS analyses

Off-line gray-scale and VH-IVUS analyses were performed using echoPlaque 4.0 software (INDEC Systems, Inc., Los Altos, CA, USA). Corresponding proximal and distal reference IVUS images were identified for each culprit lesion. These were analyzed to determine the plaque volume within the involved arterial segment. Gray-scale IVUS analysis was performed according to the American College of Cardiology Clinical Expert Consensus Document on Standards for Acquisition, Measurement and Reporting of Intravascular Ultrasound Studies. [10] In the gray-scale conventional IVUS analysis, images were assessed for lesion length, lumen volume, plaque volume, and vessel volume. In the VH-IVUS analysis, fibrous, fibrofatty, NC, and dense calcium regions were color-coded, and estimates of the contribution of each to the volume of the entire culprit lesion were reported as percentages of the plaque volume as %NC (= the percentage of NC volume). The same investigator along with another (T.S. and K.W.) subsequently reanalyzed the images to assess the intraobserver and interobserver reproducibility of the measurements.

### Clinical laboratory measurements

Laboratory evaluations of plasma lipid and apolipoprotein levels were performed within two days before PCI. Commercial reagent kits (Determiner L TC II, Determiner L LDL-C II, Determiner L HDL-C II, and Determiner L TG II; Kyowa Medex, Tokyo, Japan) were used to analyze plasma TC, LDL-C, HDL-C, and triglyceride (TG) levels, and lipid concentrations were measured using an automated chemical analyzer (Labospect 006, Hitachi, Tokyo, Japan). Apo-B and Apo-A1 were measured by immunonephelometry, and high-sensitivity C-reactive protein (hs-CRP) levels were measured by nephelometry. Apolipoprotein levels were measured at a central clinical laboratory (SRL, Inc., Tokyo, Japan).

### Statistical analysis

Data are presented as means ± standard error. Categorical data are presented as numbers (n) and percentages (%). Categorical and continuous variables were compared between the LAB and HAB groups using the chi-square test, Welch’s *t*-test, and Wilcoxon’s signed rank test. All statistical assessments were two-sided and evaluated at a significance level of 0.05. To investigate the relationship between the NC and each lipid in all patients (not divided into LAB and HAB groups), Pearson’s correlations between %NC and Apo-B, %NC and LDL-C, and %NC and Apo-A1 were also evaluated. To detect the amount of shared variance and the strength of the relationship between the variables of interest, multiple least square regression analysis was performed. The statistical analysis indicated a significant relationship between %NC and the 1^st^ model (model 1), which comprised seven predictive variables including Apo-B (age, male sex, smoking, statin use, hs-CRP, Apo-A1, Apo-B); and the 2^nd^ model (model 2), which comprised seven predictive variables including LDL-C (age, male sex, smoking, statin use, hs-CRP, Apo-A1, LDL-C). All statistical analyses were performed using Ekuseru-Toukei for Windows, version 1.02 (SSRI Co., Ltd., Tokyo, Japan).

## Results

The median Apo-B level in the 115 study patients was 91 mg/dL. There were 57 and 58 patients in the LAB (Apo-B ≤90 mg/dL) and HAB (Apo-B ≥ 91 mg/dL) groups, respectively. Antiplatelet and statin treatments were more frequent in the LAB group than in the HAB group (Table 1). Compared with the HAB group, the LAB group had lower TC, TG, and LDL-C levels and a higher HDL-C level (Table 1).

**Table 1:** Characteristics and laboratory data in patients with low and high plasma apolipoprotein B.

Characteristic	LAB group n = 57	HAB group n = 58	P
Age (years)	72.2 ± 1.2	67.8 ± 1.1	0.0086^❋^
Male (%)	40 (70.2)	48 (81.4)	0.1595
***Clinical Histories***			
Diabetes mellitus (%)	20 (35.1)	19 (32.2)	0.7423
Hypertension	40 (70.2)	45 (76.3)	0.4583
Dyslipidemia	33 (57.9)	36 (61.0)	0.7320
Smoking	14 (24.6)	26 (44.1)	0.0271^❋^
Previous PCI	29 (50.9)	23 (39.0)	0.1987
Previous Stroke	1 (1.8)	3 (5.1)	0.3257
***Oral Medications***			
CCB	30 (52.6)	28 (47.5)	0.5774
ACEI/ ARB	27 (47.4)	25 (42.4)	0.5886
Antiplatelet drugs	47 (82.5)	37 (62.7)	0.0174^❋^
Anti-DM drugs	9 (15.8)	10 (16.9)	0.8660
Insulin	1 (1.8)	1 (1.7)	0.9804
Statin	34 (59.6)	17 (28.8)	0.0008^❋^
***Culprit Vessel***			
RCA	22 (38.6)	20 (33.9)	0.5986
LAD	22 (38.6)	25 (42.4)	0.6787
LCX	13 (22.8)	14 (23.7)	0.9065
***Laboratory data***			
TC (mg/dL)	167.0 ± 3.4	212.8 ± 4.6	<0.001^❋^
TG (mg/dL)	121.7 ± 7.0	205.3 ± 12.7	<0.001^❋^
HDL-C (mg/dL)	57.1 ± 1.8	51.0 ± 1.3	0.009^❋^
LDL-C (mg/dL)	85.6 ± 2.9	121.9 ± 4.2	<0.001^❋^
Apo-A1 (mg/dL)	134.9± 2.8	123.7± 2.4	0.5569
Apo-B (mg/dL)	75.8 ± 1.4	110.2 ± 2.6	<0.001^❋^
HbA1c (%)	6.0 ± 0.1	6.2 ± 0.1	0.2526
hs-CRP (ng/mL)	1340.8 ± 427.4	1903.9 ± 431.2	0.3556

Values are means ± standard error or numbers of patients (percentage). LAB, low apolipoprotein-B (≤ 90 mg/dL); HAB, high apolipoprotein B (≥ 91 mg/dL); PCI, percutaneous coronary intervention; CCB, calcium-channel blocker; ACEI/ARB, angiotensin-converting enzyme inhibitor and angiotensin II receptor blocker; DM, diabetes mellitus; RCA, right coronary artery; LAD, left anterior descending coronary artery; LCX, left circumflex coronary artery; TC, total cholesterol; HDL-C, high-density lipoprotein cholesterol; LDL-C, low-density lipoprotein cholesterol; Apo-A1, apolipoprotein A1; Apo-B, apolipoprotein B; HbA1c, hemoglobin A1c; hs-CRP, high-sensitivity C-reactive protein.

^❋^P<0.05.

**Fig 1:**
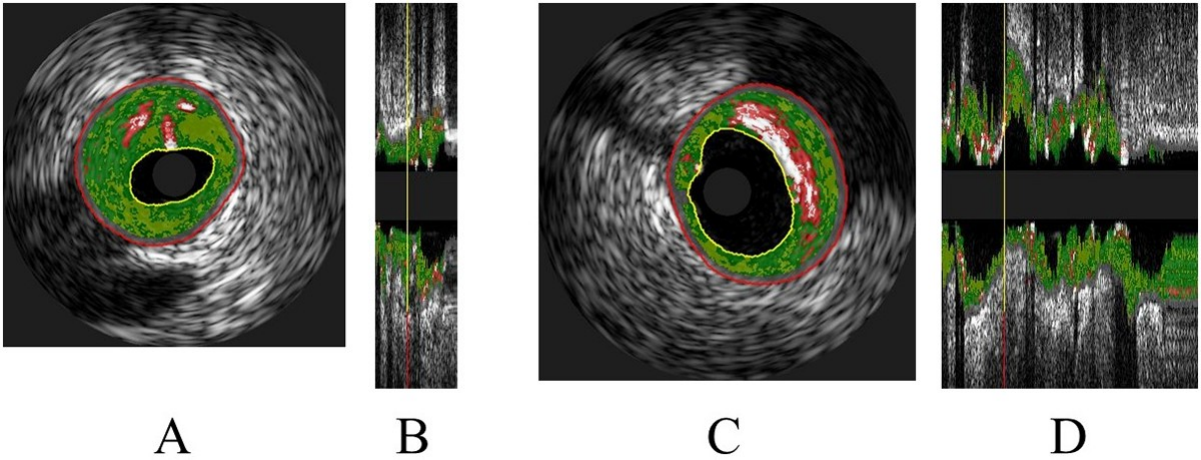
Representative virtual-histology intravascular ultrasound images. **A**. A cross-sectional image of the lesion in a patient in the low apolipoprotein B (LAB) group. The composition of the entire lesion by volume percentage is fibrous (green), 54.43%; fibrofatty (light green), 29.09%; necrotic core (red), 12.96%; and dense calcium (white), 3.52% **B**. Longitudinal section of the lesion in the same patient in the LAB group. The yellow line indicates the location of the cross-section shown in (A). **C**. A cross-sectional image of the lesion in a patient in the high apolipoprotein B (HAB) group. The composition of the entire lesion by volume percentages are as follows: fibrous (green), 48.99%; fibrofatty (light green), 20.24%; necrotic core (red), 22.36%; and dense calcium (white), 8.41%. **D**. Longitudinal section of the lesion in the same patient in the HAB group. The yellow line indicates the location of the cross-section shown in (C).

**Fig 2:**
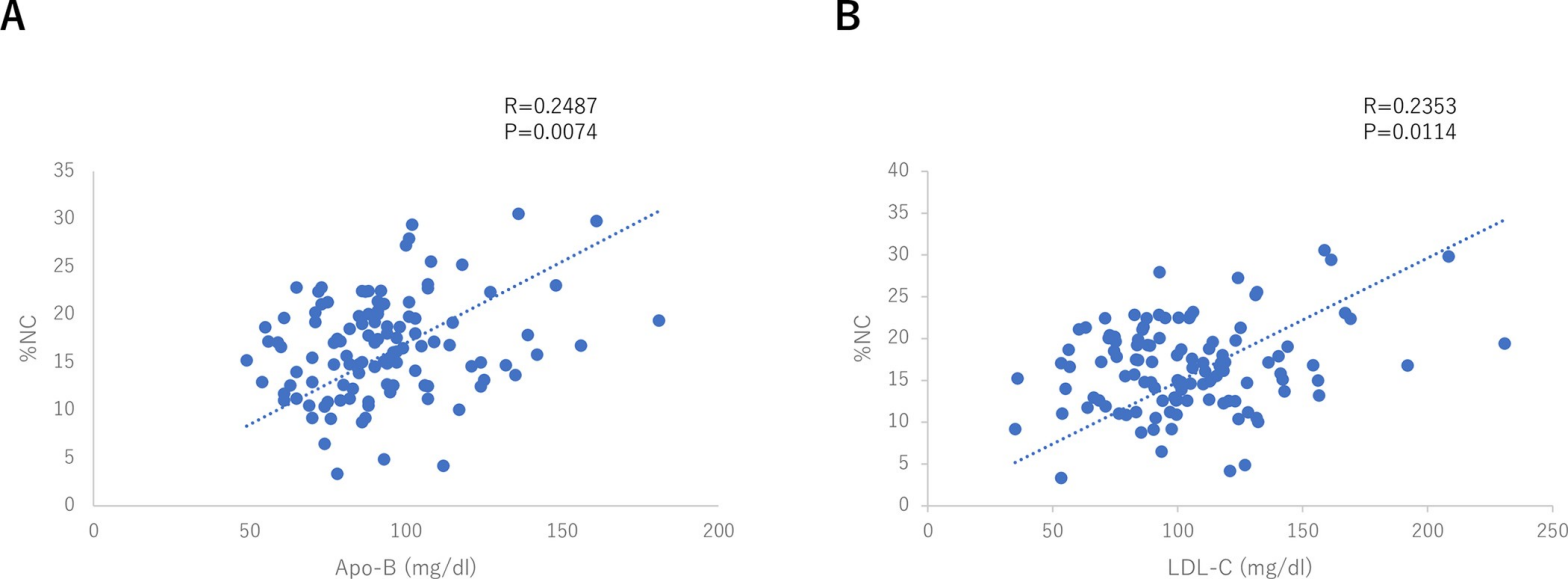
Correlation between %NC volume and Apo-B level and LDL-C level in all patients (n = 115). A. Correlation between %NC volume and Apo-B level in all patients. The %NC volume was significantly correlated with Apo-B level (r = 0.2487, P = 0.0074). B. Correlation between %NC volume and LDL-C level in all patients. The %NC volume was significantly correlated with LDL-C level (r = 0.2353, P = 0.0114). %NC, the percentage of necrotic core volume; Apo-B, apolipoprotein B; LDL-C, low-density lipoprotein cholesterol.

Representative VH-IVUS images are shown in Fig 1. The intraobserver (r = 0.98, 0.98, and 0.99) and interobserver (r = 0.96, 0.97, and 0.98) measurement variabilities were acceptable. The vessel, plaque, and %NC volumes and lesion length were significantly greater in the HAB group than in the LAB group (Table 2). In all patients, the %NC volume was significantly correlated with the Apo-B (r = 0.2487, P = 0.0074, Fig 2A) and LDL-C (r = 0.2353, P = 0.0114, Fig 2B) levels, but not with the HDL-C (r = -0.0019, P = 0.9837) and the Apo A-1 (r = 0.01, P = 0.9169) levels. The hs-CRP level tended to be higher in the HAB group (1883.356 ± 424.334) than in the LAB group (1340.807 ± 427.359, P = 0.369). However, in all patients (n = 115), the hs-CRP level was not correlated with the %NC volume (r = 0.0755, P = 0.4231).

**Table 2:** Gray scale- and VH-IVUS data in patients with low and high plasma apolipoprotein B.

	LAB group n = 57	HAB group n = 58	P
***Gray scale-IVUS data***			
Vessel volume (mm^3^)	665.2 ± 59.1	1000.1 ± 79.7	0.0011^❋^
Plaque volume (mm^3^)	399.1 ± 37.1	621.3 ± 52.4	0.0008^❋^
Lumen volume (mm^3^)	266.1 ± 23.4	378.8 ± 31.3	0.0048^❋^
Lesion length (mm)	50.9 ± 3.8	72.5 ± 4.3	0.0003^❋^
***VH-IVUS data***			
Fibrous volume (mm^3^)	140.602 ± 13.853	237.805 ± 23.030	0.0001^❋^
Fibrous volume (%)	54.400 ± 0.860	55.134 ± 0.764	0.5241
Fibrous fatty volume (mm^3^)	68.951 ± 9.570	92.804 ± 12.697	0.0347^❋^
Fibrous fatty volume (%)	23.648 ± 1.053	21.113 ± 1.083	0.0963
Necrotic core volume (mm^3^)	38.838 ± 3.830	74.304 ± 7.463	<0.00001^❋^
Necrotic core volume (%)	15.293 ± 0.608	17.622 ± 0.725	0.0157^❋^
Dense calcium volume (mm^3^)	14.702 ± 1.244	23.013 ± 2.206	0.0111^❋^
Dense calcium volume (%)	6.659 ± 0.496	6.132 ± 0.469	0.4414

Values are means ± standard error. LAB, low apolipoprotein B (≤ 90 mg/dL); HAB, high apolipoprotein B (≥ 91 mg/dL); VH-IVUS, virtual histology intravascular ultrasound.

*P<0.05.

Table 3 displays a model summary of the multiple regression analysis of the value of Apo-B as a biomarker. The value of the correlation coefficient adjusted R-square (0.9110) indicates the presence of a strong correlation between the combinations of independent variables and the %NC volume, as the level of significance is less than 0.05. In order to identify significant independent variables, we examined the values of the regression coefficients. The analysis indicated the existence of a positive relationship between male sex (coefficient: 2.2405, beta = 0.112, P = 0.039), Apo-A1 (coefficient: 0.0615, beta = 0.480, P <0.001), and Apo-B (coefficient: 0.0698, beta = 0.3866, P <0.001) and %NC volume. In order to rank the independent variables, their beta values were examined. The significance ranking was 1: Apo-A1, beta = 0.4800; 2: Apo-B, beta = 0.3866; and 3: male sex, beta = 0.1124.

**Table 3:** Model 1. Multiple regression analysis to analyze the contribution of each predictor including Apo-B in predicting the %NC (n = 115).

Predictors	B	Beta	T	P	F
male	2.2405	0.1124	2.0882	0.0390*	4.3607
Apo-A1	0.0615	0.4800	4.7806	<0.001*	22.8545
Apo-B	0.0698	0.3866	3.6008	<0.001*	12.9659

*P<0.05

Table 4 displays a model summary of the multiple regression analysis for the value of LDL-C as a biomarker. The value of the correlation coefficient adjusted R-square (0.0846) indicates the presence of a weak significant correlation between the combinations of independent variables and %NC. This indicated the existence of a positive relationship between age (coefficient: -0.1256, beta = -0.2135, P = 0.0192) and LDL-C (coefficient: 0.0347, beta = 0.2205, P = 0.0156).

**Table 4:** Model 2. Multiple regression analysis to analyze the contribution of each predictor including LDL-C in predicting the %NC (n = 115).

Predictors	B	Beta	t	P	F
Age	-0.1256	-0.2135	-2.3765	0.0192*	5.6478
LDL-C	0.0347	0.2205	2.4548	0.0156*	6.0262

*P<0.05

## Discussion

In the present study, we demonstrated that the lesion length and vessel, plaque, and %NC volumes were higher in patients with high plasma Apo-B levels than in those with low plasma Apo-B levels. Interestingly, the Apo-B (r = 0.2483) and LDL-C (r = 0.2105) levels were positively correlated with %NC volume in the VH-IVUS volumetric analyses. In this study, Apo-B and %NC appeared to be more closely correlated than were LDL-C and %NC. However, we found no correlation between Apo-A1 and %NC. Furthermore, although hs-CRP tended to be higher in the HAB group than in the LAB group, this difference was not statistically significant. On the other hand, multiple regression analysis (model 1) revealed that the male sex, Apo-A1, and Apo-B were significant predictors of %NC volume. It is interesting that Apo-A1 was not correlated with %NC volume on Pearson’s correlation analysis but affected it on the regression analysis. This can be explained by the fact that the significance of Apo-A1 in the model is dependent upon all the factors being in the model. Male sex and Apo-B were acting as a sort of mediator of the relationship between Apo-A1 and %NC volume. Moreover, these results showed that Apo-B induces plaque progression, vascular remodeling, and, especially, NC progression, the latter of which is also affected by male sex and Apo-A1. Additionally, Apo-B might be involved in plaque vulnerability. Furthermore, in model 2 of the regression analysis, age and LDL-C were significant predictors of %NC volume although the value of the correlation coefficient adjusted R-square (0.0846) was very low. On the other hand, the value of the correlation coefficient adjusted R-square (0.9110) of model 1 including Apo-B was very high, and %NC in the lesion plaque of SCD patients could be predicted by this model.

A previous study demonstrated that the accuracy of VH-IVUS for ex vivo tissue characterization exceeds 93.5%.[11] When not influenced by calcium-induced acoustic attenuation, 45 MHz VH-IVUS technology allowed tissue-type identification in combined tissues with accuracy >88%, which was greater than the accuracy of the gold standard histologic assessment, and maintained high interobserver and intraobserver reproducibility. [12] Moreover, the concordance between the histological and VH-IVUS classifications of carotid plaques was 86.1%.[13] On the basis of these reports, the accuracy of VH-IVUS plaque characterization is acceptable.

Numerous previous studies have evaluated the use of plasma biomarkers for the identification of high-risk lesions or vulnerable plaques in patients with SCD. Furthermore, VH-IVUS has frequently been used to obtain detailed information about the composition and characteristics of coronary atherosclerotic plaques. Consequently, several plasma biomarkers[14] and the plaque composition assessed by VH-IVUS [15, 16] have been proposed as predictors of plaque rupture.

Compared with LDL-C, non-HDL-C and Apo-B more successfully predict cardiovascular disease (CVD) risk. [17] The AMORIS study [18], which compared Apo-B and LDL-C, found that Apo-B was a more accurate predictor of CVD risk in 175,553 healthy individuals. Our finding that Apo-B is more strongly correlated with %NC than LDL-C is consistent with the results of these previous clinical studies.

Of note, Moss et al. [19] reported that Apo-B (hazard ratio, 1.82; 95% CI, 1.10–3.00) and low levels of Apo-A1 (hazard ratio, 1.84; 95% CI, 1.10–3.08) were independently associated with recurrent coronary events, whereas LDL-C was not. This is consistent with our finding of a correlation between Apo-B and Apo-A1 levels and %NC.

McGill et al. [20] were the first to describe the correlation between Apo-B and angiographic lesion morphology in patients with premature coronary heart disease. They demonstrated that the Apo-B level independently predicted the extent of angiographically defined coronary atherosclerosis. Moreover, the BECAIT trial [21] reported that the HDL3 cholesterol and plasma Apo-B concentrations were independent predictors of the mean minimum lumen diameter changes (r = -0.23, p < 0.05) and percent stenosis (r = 0.30, p < 0.01), respectively. While Golshahi et al. [22] demonstrated that Apo-A1 was associated with mild (but not severe) coronary atherosclerosis, they found that Apo-B was associated with severe (but not mild) coronary atherosclerosis. Further, HDL-C, LDL-C, and TG were not significant risk factors in their study.

Elevated hs-CRP is a surrogate marker of the inflammatory process that is reportedly related to the plaque burden in non-culprit coronary arteries [23] and the NC volume in cross-sections of culprit lesions in patients with stable angina. [16] In our study, hs-CRP was not correlated with %NC in the culprit lesion. Although hs-CRP levels in the HAB group tended to be higher than those in the LAB group, this difference was not significant. This difference in hs-CRP between previous studies and our own is likely attributable to the differences in methodology used. Our data were based on an estimate of the volume of the whole lesion, whereas previous studies assessed the cross-sectional area of the lesion with the smallest stenosis degree that was most affected by inflammation. However, we found that Apo-B was significantly correlated with %NC volume, and affected %NC volume on multiple regression analysis. Our data showed that, compared with hs-CRP, Apo-B was a more sensitive marker of unstable plaques with a large NC.

Fluorescein angioscopy and microscopy images of human coronary artery plaques [24] showed that Apo-B begins to deposit before plaque formation, accumulates with plaque growth, and disappears after NC formation. These results, taken together with our own, indicate that Apo-B contributes to NC formation.

The present study has some limitations worth noting. First, this was a single-center study, and the study sample was relatively small. Therefore, our results need to be validated by larger studies. Second, follow-up data were not available. Therefore, we could not assess the prognostic implications of an elevated Apo-B level in patients with SCD. Despite these limitations, our study identified a correlation between the Apo-B level and plaque composition of the target coronary artery lesion in patients with SCD.

In conclusion, an elevated Apo-B level was related to the %NC of the target coronary artery lesion in patients with SCD. Our findings suggest a potential role for Apo-B as a biomarker of unstable plaques in this patient population. Moreover, our study showed the Apo-B more strongly related to plaque advancement, and therefore is a more important contributing factor to the development of acute coronary syndrome than LDL-C. Therefore, future studies should further evaluate the role of Apo-B in cardiovascular disease pathogenesis.

## Supporting information

S1 File All individual patient data used in the study. (XLSX)
